# Segmental speech error data elicited at prosodically-defined locations in tongue twisters

**DOI:** 10.1016/j.dib.2018.07.068

**Published:** 2018-08-01

**Authors:** Mary-Beth Beirne, Karen Croot

**Affiliations:** School of Psychology, University of Sydney, Australia

## Abstract

Tongue twister error data described here were collected from 44 unimpaired speakers producing six repetitions each of 40 tongue-twisters that manipulated the position of error-prone segments within two prosodic domains: intonational phrases and utterances. Data are counts of perseveration and anticipation errors on the initial segments of phrase-initial words or phrase-final words. The location of the phrase-level prominence and other factors associated with speech errors were carefully controlled. For more details about the design, materials and methods, and for interpretation and discussion, see Beirne and Croot (2018) [1].

**Specifications Table**

**Table:** 

Subject area	*Psychology, Linguistics, Speech Science*
More specific subject area	*Speech production*
Type of data	*Excel workbook*
How data was acquired	*Speech errors were transcribed from experimentally elicited tongue twisters produced by unimpaired speakers*
Data format	*Counts of speech errors*
Experimental factors	*Speech errors were transcribed from audio recordings and categorized as perseveration or anticipation errors*
Experimental features	*Participants produced six repetitions each of two-intonational-phrase tongue-twisters with error-prone word-initial segments in the phrase-initial and phrase-final words. Confounding factors were controlled.*
Data source location	*University of Sydney, Sydney, Australia*
Data accessibility	*Data are provided with this article in Excel workbook entitled:* Beirne_Croot_Data_in_Brief_2018.xlsx
Related research article	*Beirne, M.B. & Croot, K.*[1]

**Value of the data**

• Tongue twister errors elicited in generally meaningful connected speech are available for comparison with similar data elicited in lists.
• The tongue twisters in which these data were elicited control the location of the phrase-level prominence such that it does not fall on the tongue twister words, allowing comparison with errors elicited in tongue twisters with other prosodic structures.
• The tongue twister errors were collected using impressionistic broad phonetic transcription, allowing comparison with errors observed using other measurement techniques.

## 1 Data

Data are counts of segmental speech errors presented in four worksheets within an excel workbook according to whether they were produced in the phrase-initial or phrase-final condition, and whether the rows show participants or items.

## 2 Experimental design, materials and methods

The tongue twister error data reported here were collected from 44 undergraduate psychology students with English (typically Australian English) as their first language (12 male, mean age = 19.9, range 17–45).

The experimental stimuli were 40 tongue-twister items, each composed of two syntactically well-formed 5-word clauses, designed to be elicited with the prosodic structure of two intonational phrases within an utterance. Items were generally meaningful, with some semantic anomalies. Each intended intonational phrase (henceforth, simply, “phrase”) contained a set of four monosyllabic, singleton onset “tongue-twister words,” and a number or function word. Tongue-twister words in each item began with a “target segment” (henceforth ‘B’), or one of two “confusable segments” (‘A’ and ‘C’) that were equally likely to participate in an error with B [2]. Two words beginning with A and two beginning with B were presented in alternating order in one phrase of the utterance, and two words beginning with C and two additional words beginning with B were presented in alternating order in the other phrase. In half the items the target segment B occurred at the beginning of the initial word in the phrase: this was the Phrase-Initial word condition. In the other half of the items, B occurred at the beginning of the final word in the phrase: this was the Phrase-final word condition.

The function word or number included in each phrase had initial segments unlikely to participate in errors with the A, B or C segments [2]. Lower error rates have been observed on syllables carrying narrow informational focus in intonational phrases [3], so to prevent the phrase-level prominence confounding error rates associated with the Phrase-initial or Phrase-final word positions, one of the number/function words in each item was elicited with narrow informational focus. Participants were asked to say each tongue twister item aloud as naturally as possible in response to a given question, emphasising the number/function word that would answer the question. Errors on the target segment in the intonational phrase containing the prominent number/function word were analysed. The order of initial segment pairs within phrases, the order of phrases within utterances, and the order of the phrase containing the prominent number/function word, were all counterbalanced across participants. For examples, see Figure 1 in [1]. A list of all items is given in Appendix 1 in [1], where the numbering of items is the same as shown in the by-items data here.

Participants read each tongue twister item aloud six times in succession at an approximately constant speech rate guided by a metronome of 160 beats per minute (BPM). Responses were recorded and errors were transcribed from the recordings using broad phonetic transcription.

Errors were substitutions of a target segment by one of the confusable segments, additions of a confusable segment including segments that were doubly-articulated with the target, or part of a larger error unit. They were self-corrected, or corrected-to-error, and occurred in completed and incomplete words. If an error on the target segment B had its source on the initial segment of preceding confusable word, it was classed as a perseveration error. If the source was the initial segment of the following word, it was classed as an anticipation error. Tongue twister words within items were matched on median frequency and mean neighbourhood density [4] and were controlled for lexical bias (the likelihood of errors resulting in words more often than non-words [5], [6]) and for the number of anticipatory and perseveratory errors resulting in words across phrase and utterance boundaries. Errors on Phrase-initial words in Repetitions 2–6 and on Phrase-final words in Repetitions 1–5 were analysed, with Repetition 1 or 6 discarded respectively to match the opportunities for anticipation and perseveration errors within repetitions within items.

Note that there is an inconsistency in the data in the number of errors contained in the Phrase-initial error spreadsheet arranged by participants compared with the Phrase-initial error spreadsheet arranged by items. The inconsistency lies in the cell measuring anticipation errors in Phrase 1 on Repetition 6, where the by-participants spreadsheet shows 4 errors and the by-items spreadsheet shows 5 errors. This inconsistency is present back through our earliest versions of the data and we are unable to further determine which count is correct and which incorrect. There were 466 or 465 errors in total: 79 or 80 in the phrase-initial condition, 386 in the phrase-final condition.

For more details about the design, materials and methods, see [1].
